# Invasive *Candida* Infections in Neonates after Major Surgery: Current Evidence and New Directions

**DOI:** 10.3390/pathogens10030319

**Published:** 2021-03-09

**Authors:** Domenico Umberto De Rose, Alessandra Santisi, Maria Paola Ronchetti, Ludovica Martini, Lisa Serafini, Pasqua Betta, Marzia Maino, Francesco Cavigioli, Ilaria Cocchi, Lorenza Pugni, Elvira Bonanno, Chryssoula Tzialla, Mario Giuffrè, Jenny Bua, Benedetta Della Torre, Giovanna Nardella, Danila Mazzeo, Paolo Manzoni, Andrea Dotta, Pietro Bagolan, Cinzia Auriti, on behalf of Study Group of Neonatal Infectious Diseases

**Affiliations:** 1 Neonatal Intensive Care Unit, Medical and Surgical Department of Fetus, Newborn and Infant, “Bambino Gesù” Children’s Hospital IRCCS, 00165 Rome, Italy; domenico.derose@opbg.net (D.U.D.R.); alessandra.santisi@opbg.net (A.S.); mariapaola.ronchetti@opbg.net (M.P.R.); ludovica.martini@opbg.net (L.M.); andrea.dotta@opbg.net (A.D.); 2 Neonatal Intensive Care Unit, Department of Critical Care Medicine, “A. Meyer” University Children’s Hospital, 50139 Florence, Italy; lisa.serafini@meyer.it; 3 Neonatology Unit, Azienda Ospedaliero-Universitaria “Policlinico-Vittorio Emanuele”, 95124 Catania, Italy; mlbetta@yahoo.it; 4 Neonatal Intensive Care Unit, Giovanni XXIII Hospital, 24127 Bergamo, Italy; mmaino@asst-pg23.it; 5 Neonatology Unit, Ospedale dei Bambini “V. Buzzi”, ASST FBF-Sacco-Buzzi, 20154 Milan, Italy; francesco.cavigioli@asst-fbf-sacco.it (F.C.); ilaria.cocchi88@gmail.com (I.C.); 6 Neonatal Intensive Care Unit, Fondazione IRCCS Ca’ Granda Ospedale Maggiore Policlinico, 20122 Milan, Italy; lorenza.pugni@mangiagalli.it; 7 Neonatology Unit, Azienda Ospedaliera SS. Annunziata, 87100 Cosenza, Italy; elvirabonanno@libero.it; 8 Neonatal Unit and Neonatal Intensive Care Unit, Fondazione IRCCS Policlinico San Matteo, 27100 Pavia, Italy; c.tzialla@smatteo.pv.it; 9 Department of Health Promotion Sciences, Maternal and Infant Care, Internal Medicine and Medical Specialties “G. D’Alessandro”, University of Palermo, 90133 Palermo, Italy; mario.giuffre@unipa.it; 10 Neonatal Intensive Care Unit, Institute for Maternal and Child Health IRCCS “Burlo Garofolo”, 34137 Trieste, Italy; jenny.bua@burlo.trieste.it; 11 Neonatal Intensive Care Unit, Santa Maria della Misericordia Hospital, 06123 Perugia, Italy; benedetta.dellatorre@ospedale.perugia.it; 12 Division of Neonatology, Azienda Ospedaliero-Universitaria “Ospedali Riuniti”, 71122 Foggia, Italy; giovannanardella@yahoo.it; 13 Neonatology Unit, Policlinico Gaetano Martino, 98124 Messina, Italy; danilamazzeo@outlook.it; 14 Division of Pediatrics and Neonatology, Department of Maternal, Neonatal, and Infant Health, Ospedale degli Infermi, ASL Biella, 13875 Ponderano, Biella, Italy; paolomanzoni@hotmail.com; 15 Neonatal Surgery Unit, Medical and Surgical Department of Fetus, Newborn and Infant, “Bambino Gesù” Children’s Hospital IRCCS, 00165 Rome, Italy; pietro.bagolan@opbg.net

**Keywords:** invasive *Candida* infections, invasive fungal infections, antifungal prophylaxis, newborns, surgery, neonatal surgery

## Abstract

Infections represent a serious health problem in neonates. Invasive *Candida* infections (ICIs) are still a leading cause of mortality and morbidity in neonatal intensive care units (NICUs). Infants hospitalized in NICUs are at high risk of ICIs, because of several risk factors: broad spectrum antibiotic treatments, central catheters and other invasive devices, fungal colonization, and impaired immune responses. In this review we summarize 19 published studies which provide the prevalence of previous surgery in neonates with invasive *Candida* infections. We also provide an overview of risk factors for ICIs after major surgery, fungal colonization, and innate defense mechanisms against fungi, as well as the roles of different *Candida* spp., the epidemiology and costs of ICIs, diagnosis of ICIs, and antifungal prophylaxis and treatment.

## 1. Introduction

Yeasts are commensal microorganisms that usually colonize mucosal surfaces and skin. In particular clinical conditions, such as immune suppression, prolonged use of broad-spectrum antibiotics and/or steroids, the balance of the colonizing flora of the skin is altered and fungi express numerous factors that contribute to pathogenicity. Adherence is one of the most important factors that facilitate the colonization and dissemination of fungi, by the expression of adhesins, which facilitate binding to host substrates, including beta–integrins, on the endothelium and white blood cells. The yeast-to-hypha transition of *C. albicans* facilitates biofilm formation, tissue invasion, and dissemination of the infection [1,2]. Other virulence factors are membrane and cell wall barriers, dimorphism, biofilm formation, signal transduction pathway, proteins related to stress tolerance, hydrolytic enzymes (e.g., proteases, lipases, hemolysins), and toxin production) [3].

Therefore, fungi can lead to severe infections in the host. Invasive fungal infections (IFIs) in neonatal intensive care units (NICUs) are a substantial health problem, as they are the second most common cause of infection-related death among critically ill neonates. IFIs lead also to significant neurodevelopmental disability among survivors, representing a substantial health problem, especially among the neonates with lowest gestational age and lowest birthweight [4,5,6]. Critically ill patients in NICUs (and in particular preterm neonates) are at high risk of IFIs, especially if they need broad-spectrum antibiotic treatments, surgery that disrupts natural defense barriers, intravascular catheters for prolonged periods, or implantation of invasive devices to survive. Their immunological impairments are predisposed to fungal colonization and to subsequent systemic infection. Bloodstream infections due to the *Candida* species (*C.* spp.) are considered the most common IFIs in critically ill patients in NICUs.

In specific subgroups (e.g., abdominal surgical patients), IFIs are also frequent, but there are no epidemiological studies on the incidence of IFIs in neonates with major surgical diseases. Clinical and epidemiological studies are needed to identify preventive strategies in preterm and term infants, who undergo major surgery or specific subgroups of this category of patients.

This review aims to summarize scientific evidence about invasive *Candida* infections (ICIs) in neonates undergoing surgery.

## 2. Methods

This paper provides a review of the literature on ICIs in neonates after major surgery. An extensive literature search in the MEDLINE database (via PubMed) has been performed from 2000 up to 9 January 2021. The following keywords “*Candida*” OR “fungal infection” AND “surgery” AND “neonates” OR “infants” were searched as entry terms. All retrieved articles were screened, and then full texts of records deemed eligible for inclusion were assessed. References in the relevant papers were also reviewed.

Papers written in languages other than English, or not providing data about the main focus of this research (the number of neonates with ICIs after undergoing major surgery, separate data for neonates and children, and case reports and reviews) were excluded.

Major surgery is considered to be any invasive operative procedure in which a mesenchymal barrier is opened (pleural cavity, peritoneum, meninges).

An infant is considered colonized by *Candida* when a surveillance culture (such as pharyngeal or tracheal swab, urine, feces, skin swabs) develops colonies of *Candida* spp., without signs or symptoms suggestive of infection [7].

Infants with ICIs have specific or nonspecific signs or symptoms, and isolation of a *Candida* spp. is obtained from a sterile cultural site (blood, cerebrospinal fluid, peritoneal fluid) [8].

We also provided an overview of risk factors for ICIs after major surgery, the innate defense mechanisms against fungi, the role of different *Candida* spp., the epidemiology and costs of ICIs, and the antifungal prophylaxis.

## 3. Results

A total of 253 records were identified through literature search (via PubMed) from 2000 onwards. Among them 155 were excluded based on the titles, the abstracts, and the type (case reports and review). The remaining 71 full-text articles were assessed for eligibility. We found no studies focused on the incidence of IFIs in neonates who previously underwent major surgery.

**Figure 1:**
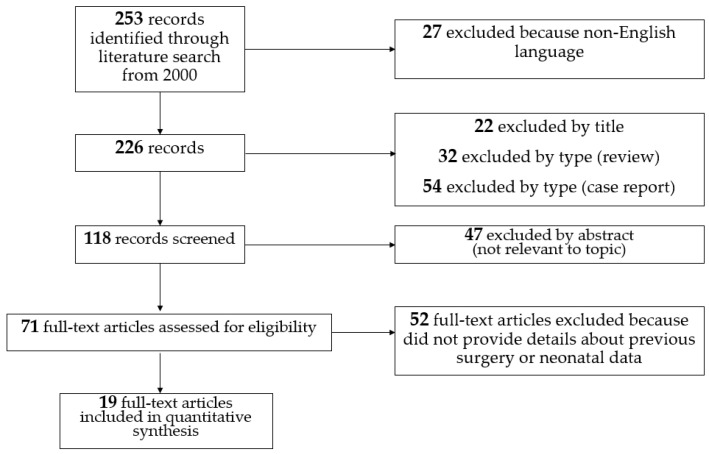
Literature selection of recent studies reporting incidence of previous surgery in infants with invasive fungal infections.

Conversely, we found 19 studies that provided how many neonates with IFIs underwent major surgery before the onset of the infection [9,10,11,12,13,14,15,16,17,18,19,20,21,22,23,24,25,26,27]. The selection process is shown in Figure 1.

Of the 19 studies included in the quantitative synthesis, 8 collected patients’ data retrospectively, while 11 collected it prospectively (Table 1). A total of 1637 neonates with IFIs were reported. Of these, 550 (33.6%) underwent major surgery before the onset of the infection. Abdominal surgery was not always reported by the studies, with percentages ranging from 13 up to 80. Fungal-infection-related mortality is difficult to demonstrate, therefore in-hospital overall mortality is more often reported.

## 4. Risk Factors for Invasive *Candida* Infections after Major Surgery

**Figure 2:**
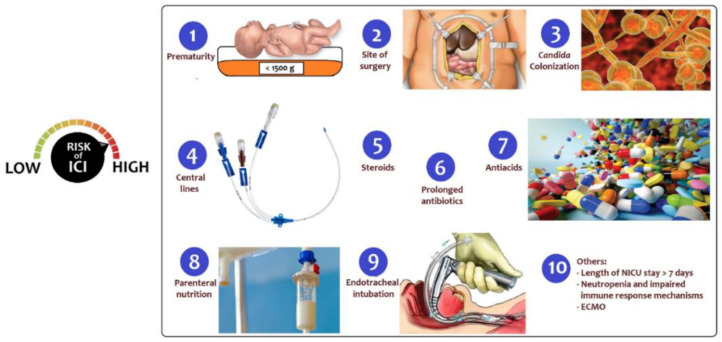
Risk factors for invasive *Candida* infections after major surgery.

Infants in NICUs for surgical diseases are at high risk for IFIs, as a result of prematurity, the need for invasive procedures, the disruption of natural barriers due to surgery and other many risk factors (Figure 2) [28,29]. Bloodstream infection due to *Candida* spp. is considered the most common IFI in critically ill patients [30,31,32,33].

Well-recognized risk factors associated with ICIs are:

1. **Prematurity**: Prematurity was recognized as the most common underlying condition (78%) among newborns with candidemia, with a median gestational age at birth of 25 weeks (IQR: 24–26) according to the United States’ Centers for Disease Control and Prevention’s (CDC’s) active population-based surveillance [22]. Most preterm neonates had a very low birth weight (VLBW, 1000–1500 g) or extremely low birth weight (ELBW, <1000 g) [22]. Mortality is high in ELBW infants with ICIs: Benjamin et al. reported an overall mortality of 34% for ELBW infants with ICIs compared with 14% for ELBW infants without ICIs [34].
2. **Site of surgery:** Surgery in the 90 days before diagnosis was the most common (38%) underlying condition among infants with ICIs. The abdomen was the most common site of surgery, according to data from four US CDCs [22]. Gastrointestinal diseases, including congenital anomalies (i.e., gastroschisis, omphalocele, duodenal or ileocolic atresia/stenosis, necrotizing enterocolitis with intestinal perforation, stoma carriers in any location) predispose patients to candidemia, as a result of a compromised intestinal barrier that promotes translocation of *Candida* colonizing the gastrointestinal tract [35].
3. ***Candida* colonization**: *Candida* colonization is the most important risk factor for ICIs and is further discussed below; it can involve from 10% to 60% of preterm babies during their hospital stay in NICU [36].
4. **Use of central lines**: Despite numerous efforts in recent decades to reduce the incidence of central line associated sepsis (CLABSI) and central lines related sepsis (CRBSI) in NICUs, such infections still represent a major complication of health care assistance in those critically ill infants. Central-line-associated blood-stream infections (CLABSIs) arise from at least 48 h after CVC insertion to 48 h after CVC removal. Catheter-related blood-stream infections (CRBSIs) are bacteremias with positivity of CVC blood cultures developing at least 2 h earlier compared to peripheral blood cultures, or when the same organism is recovered from percutaneous blood culture and catheter lumen blood culture, with 3-fold greater colony count in the latter [37]. In particular, newborns undergoing major surgery in most cases have a central vascular catheter and are most susceptible to these infections. Among the germs involved in the genesis of CLABSIs, *Candida* spp. represented the third most common pathogens (13%), after *Coagulase-Negative Staphylococci* (28%), and *Staphylococcus aureus* (19%) in a study in 304 NICUs [38]. The length of stay of indwelling catheters is a strong risk factor for CLABSI and CRBSI, while no differences have been reported between the CLABSI incidence in femoral vein catheters, peripherally inserted catheters, and umbilical venous catheters [38]. Catheter removal is recommended if a CRBSI caused by *coagulase-negative Staphylococci*, gram-negative bacilli (*Pseudomonas aeruginosa* and *Klebsiella pneumoniae*), and fungi occurs, due to the particular ability of these germs to form an intraluminal biofilm, resistant to antibiotics and/or antifungals. Biofilms on indwelling catheters may be composed of gram-positive or gram-negative bacteria or yeasts. It consists of microbial cells surrounded by a self-secreting polymer matrix, that is released into the extracellular space [39]. The matrix is composed of water, polysaccharides, proteins, lipids, and extracellular DNA. This matrix provides a protective barrier from the surrounding environment and is able to hinder the penetration of antimicrobial drugs, while also providing protection against the host’s immune defense mechanisms. From this biofilm, germs are progressively released, causing the infection to persist and favoring the dissemination of microbes to additional sites in the body. The biofilm is very difficult to eradicate from the catheter, due to the difficult penetration of antimicrobial drugs into the matrix. Therefore, CVC removal is the gold standard approach in cases of CRBSI that do not respond to systemic treatment [37,40]. The best timing of central venous catheter removal in the presence of an associated and/or catheter-related *Candida* infection has been studied by many authors [40,41], which demonstrated that early catheter removal in candidemia is associated with better outcomes in terms of shorter duration of infection, reduced mortality, and reduced long-term neurologic disabilities. When catheter removal is not recommended for the patient’s condition, the lock therapy with antimicrobials may be an option. This rescue therapy has shown promise as a strategy for the treatment of CRBSI due to several *Candida* species. The most promising strategies of antifungal lock therapy include the use of amphotericin B, ethanol, or echinocandins [42,43].
5. **Use of corticosteroids**: Treatment with corticosteroids is a risk factors for invasive fungal infections in the neonatal period. However, data are controversial. The addition of steroids to the antibiotic therapy in animal models increases the intestinal colonization, with an increase in the incidence of invasive infections [44]. Yu et al. reported no significant differences between neonates with ICI and their control peers reviewing medical charts of 5135 NICU admissions [17]. Length and dosage of steroid treatment may play a role in altering the risk in these infants.
6. **Use of prolonged broad-spectrum antibiotics**: Longer duration of antibiotic treatment, in particular third-generation cephalosporins, vancomycin, or carbapenems, increases the risk of ICIs [17]. One of the hypotheses for the role of cephalosporins is that their concentration within the biliary system would cause intestinal dysmicrobism, favoring the proliferation of opportunistic germs, in particular fungi. Considering the antibiotic and other drugs exposure and the risk of infection by species of *Candida*, the third generation of cephalosporins seems to be a risk factor for *Candida albicans* infection, while parenteral nutrition, lipidic emulsion, and H2 antagonists are risk factors for *Candida parapsilosis* infections [11].
7. **Use of antacids**: Inhibitors of gastric acidity such as proton-pump inhibitors (PPIs, e.g., omeprazole) are widely used to prevent and manage feeding intolerance and gastroesophageal reflux, although few data on safety and efficacy are available. However, PPIs potentially increase the risk of systemic infections and necrotizing enterocolitis (NEC), especially in preterm infants [45]. In a multicenter cohort of 743 infants, the main pathogens causing infections in infants exposed to inhibitors of gastric acidity were gram-negative-bacilli and *Candida* spp. [46].
8. **Use of parenteral nutrition**: Parenteral nutrition (PN) is often considered an ideal microbial growth medium, and lipid administration in particular poses a specific risk for microbial growth [47]. PN given without the use of appropriate filters could contribute to potentially important extrinsic mechanism of infection in NICU patients [11]. Patients with species other than *C. albicans* were more likely to have PN than those with *C. albicans* (96.3% versus 71.4%, *p* = 0.039) [48].
9. **Endotracheal intubation and invasive devices**: Surgical and mechanical devices such as endotracheal tubes, drains, or urinary catheters may be also responsible for the nosocomial spread of pathogens. According to an epidemiologic surveillance study, two devices increased the relative risk for nosocomial infections by 2.6 times and three devices by 3.6 times [49].
10. **Others**: A length of stay in NICU >7 days was reported as one of the main potential risk factors by a multicenter IFI surveillance project (the AURORA project) [50]. Lack of, or inadequate, hand hygiene of healthcare workers has been also reported as one of the main reasons for horizontal transmission of virulent *Candida* spp. responsible for the invasive infections in critical patients, such as neonates [51]. Neutropenia, defined as neutrophil count <1500/mm^3^, was found as an independent predictor of candidemia in NICUs [52,53]. Extracorporeal membrane oxygenation (ECMO) procedures and locations may contribute to acquired infection risk and the most common organisms identified were *coagulase-negative Staphylococci*, followed by *Candida*, and *Pseudomonas* species at eight children’s hospitals [54].

## 5. *Candida* Colonization

The percentage of colonized preterm infants who develop invasive infections ranges from 5% to 30% [55], due to immunological immaturity, the immaturity of the skin and of mucous membranes, and to the need for invasive therapeutic supports. In general, all patients admitted to intensive care are exposed to fungal infections, some of them are effectively colonized, and only a minority develop systemic infections that originate from peripheral colonization. In intensive care units other than neonatal intensive care units (e.g., surgical intensive care units) the risk of colonization is greater in the presence of central venous catheters, bladder catheters, mechanical ventilation, and lack of enteral nutrition. Skin and the gastrointestinal tract are the most common sites for *Candida* colonization in preterm and term infants [35,56]. Colonization can take place either vertically, from the maternal genital tract, or horizontally, by transmission of the germ through the hands of health caregivers. The use of broad-spectrum antibiotic therapy favors *Candida* spp. colonization, even if it does not seem to condition the transition from colonization to systemic infection. Some researchers have shown that the addition of steroids to antibiotic therapy in animal models increases the intestinal colonization, with an increase in the incidence of invasive infections [44].

The frequency of colonization is inversely proportional to the neonate’s gestational age and birth weight. Severely preterm infants are the most affected, experiencing invasive infections following colonization. Furthermore, the risk of invasive infections is proportional to the number of colonized body sites and their localization. More noncontiguous colonization sites are associated with a greater probability of progression to invasive infection. Therefore, preterm and full-term infants colonized in more than one body site are more likely to develop invasive *Candida* spp. infection the less contiguous the colonization sites are [57]. Colonization at two or more sites occurs similarly with *Candida albicans* and *Candida parapsilosis*, while *Candida albicans* is most frequently responsible when more than two sites are involved [58].

Isolation of *Candida* spp. from the urine of neonates can be indicative of contamination or of urinary tract infection (UTI), although any positive culture from normally sterile body fluids such as urine, peritoneal fluid, or cerebrospinal fluid is often considered as an invasive candidiasis that needs to be treated as well as candidemia [5]. To date, it is still not clear how often *Candida* UTI (defined as growth of *Candida* from urine at >10^6^ CFU/L from a suprapubic aspirate or >10^7^ CFU/L from a bladder catheter specimen) is a precursor of candidemia or of *Candida* infection at other sites [59]. Among 30 infants with candiduria, an active surveillance (PICNIC study) detected 4 infants who developed extra-renal dissemination of *Candida* infection. In this study, the extra-renal site was blood in 3/4 cases and the central nervous system in 1/4; involved species were *Candida albicans* (75%) and *Candida parapsilosis* (25%) [59]. Three of these infants had a congenital heart disease and were treated between candiduria and positive culture at the extra-renal site with amphotericin B, fluconazole, or both; one was a 26-weeks preterm infant. Days between positive urine culture and positive culture at the extra-renal site ranged from 2 to 41 [59].

## 6. Innate Defense Mechanisms against *Candida* and Surgery

Innate immunity is critical for the survival of neonates, who encounter for the first time a lot of new micro-organisms, such as *C. albicans*, which is the most common fungal pathogen found in NICUs. A wide range of genetic and epigenetic factors may influence neonatal innate immunity [60]. Dysregulation of neonatal innate immune responses increase their susceptibility to severe infections [61].

Polysaccharide structures of *C. albicans* cell wall, such as β-glucans and mannans, constitute the main pathogen-associated molecular patterns (PAMPs) involved in *Candida*–host immune system interaction [62]. In the absence of a specific antibody-mediated opsonization, that cannot be mounted by neonates, PAMPs are identified by pattern-recognition receptors (PRRs) expressed on immune cells’ surfaces, as macrophage mannose receptors (MMR) and toll-like receptors (TLRs) [63]. Neonatal macrophages are capable to phagocytize *Candida* spp. using MMR, but cannot be entirely stimulated by interferon-γ (IFN-γ), considering the lack of a normal regulation of IFN-γ receptor in neonates [64].

An intact epithelium and endothelium represent important mechanical barriers against fungal invasion [62]. The formation of fungal hyphae contributes to epithelial damage and immune activation through Candidalysin, a recently discovered peptide toxin, encoded by the *ECE1* gene [65].

The intestinal mucosal barrier plays a key role in the protection against an invasion of fungal pathogens. In fact, gut cells behave not only as a physical barrier but have also an active role producing mucus and anti-microbial peptides such as β-defensins [66,67]. However, in the case of impaired barriers, *Candida*, which is usually found in the gut, may invade the intestinal epithelial barrier and translocate into the bloodstream, especially in case of abdominal surgery [67].

Furthermore, mucosal colonization by *Candida* spp. (and *C. albicans* in particular) is a major risk factor for potential life-threatening candidemia [68]. The presence of *C. albicans* stimulates the mitogen-activated protein kinase (MAPK) pathway and c-Fos activation, likely with a threshold level to activate immune response. The threshold could be pivotal in triggering an inflammatory response from a simple colonization [67].

## 7. Population Microdiversity and Role of Different Species of *Candida*

*Candida* spp. are distributed differentially according to age: *C. albicans* and *C. parapsilosis* are prevalent in neonates [69], whereas adults are mainly affected with *C. albicans* and *C. glabrata* [70]. In the largest study to date (EUROCANDY), involving 23 pediatric centres, *C. albicans* (52.5%) and *C. parapsilosis* (28%) were the predominant species, followed by *C. tropicalis, C. glabrata, C. krusei* and other rare species (including *C. dubliniensis, C. pulcherrima, C. blankii, C. famata, C. guilliermondii, C. lusitaniae, C. magnolia, C. orthopsilosis, C. zeylanoides*). *C. albicans* was prevalent among neonates (60.2%), while highest infection rates of *C. parapsilosis* were observed among infants (42%), with significantly lower prevalence in neonates (26%) [71]. Similar data were reported by a multicenter pediatric and neonatal study (involving 23 centers in the United States and 19 in 15 other countries), with 48% *C. albicans* isolates and 28% *C. parapsilosis* isolates in newborns [72]. Focusing on patients of surgical intensive care units of the EUROCANDY cohort, 72.2% episodes were due to *C. albicans* while the remaining cases were ascribed to *C. parapsilosis*. However, the number of neonates, infants, and children who underwent major surgery was not specified [71]. High-risk neonates become colonized with *Candida* spp. not only vertically during vaginal birth from their mothers, who may be receiving an azole for vaginal candidiasis, but also horizontally from colonized hospital-workers during their stay in NICU.

Although *C. albicans* remains the most common isolate in NICU, a shift to infections caused by *C. parapsilosis* and *C. tropicalis* has occurred during the last decades, and it has been associated with decreased mortality [12].

Among all ICIs, *C. albicans*, *C. parapsilosis, C. tropicalis, C. glabrata,* and *C. krusei* account for nearly 90% of isolates from blood or other sterile site cultures. Candidemia caused by other uncommon species, such as *C. guilliermondii*, and *C. lusitaniae*, is less well-known. It seems, though, to have a poorer response to antifungal treatment (frequently due to antifungal minimal inhibitory concentration -MIC- above the epidemiologic cut-off value) and a longer duration of candidemia [25].

Whereas specific inflammatory and tissue-destructive histopathologic features were found in most neonatal *C. albicans* cases, the mechanisms underlying cases of species other than *C. albicans* are still poorly understood. According to autopsy-based data, species other than *C. albicans* could involve both the gastrointestinal tract and pulmonary airways and their incidence is often underrated [73].

## 8. Epidemiology of Fungal Infections in NICUs

Although there is an inter-site variability in the incidence of candidemia [22,71], prevention of ICIs should be an achievable evidence-based goal for every NICU [74]. NICU and PICU admissions were considered as significant predictors for mortality, with an odds ratio of 4.67 and 8.325, respectively, in the EUROCANDY cohort [71]. However, most data involve extremely preterm infants.

In specific subgroups of patients (e.g., abdominal surgical patients), ICIs are also frequent [30,31,32,33,75], but to date there are no large epidemiological studies on the incidence of ICIs in neonates who have undergone major surgery. *Candida* spp., within four weeks from admission in intensive care units, colonize the skin and mucous membranes of about 64% of critically ill neonates and can progress to invasive infection [76].

ICIs are a major cause of morbidity and mortality among critically ill patients [31,77,78] and impose an important economic burden mainly due to prolonged ICU stay, cost of antifungal drugs, and overall use of hospital resources [79,80].

In case of nosocomial ICI outbreaks, a cluster of infections could be defined when at least two cases of severe neonatal infection (i.e., bloodstream infection) occur within a defined time interval in one center with the same pathogen species in different patients: *Candida albicans* is one of the most frequently occurring microorganisms, according to a recent German surveillance system [81].

Therefore, a nosocomial ICI outbreak within a NICU could have important clinical and economic repercussions. A contaminated environment has been identified as a possible source of the outbreak: the colonized locations included wiping cloths, faucets, sinks, an operating table, puddles in the bathroom, a ventilator, and an ultrasonic probe in a recent outbreak of *Candida parapsilosis* fungemia in a Chinese hospital [82]. An emergency plan should be promptly scheduled with environmental surveillance and comprehensive interventions, such as hand hygiene and disinfection techniques. Improving both disinfection and isolation, as well as interrupting the pathway of transmission, resulted to be the key to controlling the spread of infection [83].

New methods (such as fingerprinting analysis of *Candida* isolates) can help to identify the identical strains, to investigate suspected outbreaks and to help therapeutic decision-making [84].

## 9. Prophylaxis of Fungal Infections

The high-risk population of critically ill neonates benefits greatly from prompt, effective treatment and prophylactic measures. A prompt antifungal treatment is one of the most important determinants for mortality reduction. In addition, antifungal prophylaxis given to critically ill patients at high risk for ICIs may have a positive impact on patients’ outcomes, given ICIs’ high morbidity and mortality rates [85,86].

Fluconazole prophylaxis has been proven to be safe and effective in neonates, redu-cing ICIs by more than 80% and *Candida*-related mortality by 90%, especially in high-risk preterm infants, without significant side effects or emergence of resistant fungal species [75]. Considering its long half-life plasma elimination, which allows an intermittent administration schedule, fluconazole should be administered at 3 mg/kg once a day, two times a week in the first two weeks of life whereas, from the third week of life, prophylaxis should be administered every other day [4]. The benefits of prophylaxis are less clear when incidence of ICIs is lower than 2%, and the administration should be discussed case by case, in relation to the presence of risk factors for ICIs.

There is currently clear evidence on the efficacy of fluconazole prophylaxis in the prevention of ICIs in preterm infants [87,88,89,90,91], but not in surgical newborns. In these neonates, fluconazole prophylaxis is not clearly suggested, although they are considered at risk of ICI as explained above. A major concern regarding a larger prophylactic use of antifungal agents, even in term infants with risk factors, is the emergence of resistant species. However, resistance to fluconazole or echinocandins in newborns is reported as rare: fluconazole-resistant *C. albicans* was seen among 1.6% of the isolates, while no echinocandins-resistant *C. albicans* was observed [23].

## 10. Diagnosis of Invasive *Candida* Infections

Diagnosis of ICIs is very difficult in newborns, as clinical signs and symptoms of ICIs can be nonspecific and often subtle. For this clinical, radiological, and mycological evaluations should be carried out simultaneously. In addition to microbiological cultures (blood, urine, cerebrospinal fluid, peritoneal fluid, tracheal aspirate), laboratory techniques for diagnosing ICIs also include the direct microscopic examination, the histological examination of the involved tissues, the evaluation of fungal antibodies and fungal antigens (galactomannan, 1,3-beta-d-glucan) by enzyme-linked immunosorbent assay (ELISA) or by immunofluorescence, and the detection of fungal DNA by polymerase chain reaction (PCR) in blood and/or other biologic fluids. While fungi grow readily in culture media, their identification requires large volumes of blood, which are difficult to collect, especially in preterm infants. Therefore, blood cultures can be negative in a large number of patients with fungal sepsis. In addition, up to 50% of infants with positive cerebrospinal fluid (CSF) for *Candida albicans* or *Candida parapsilosis* may have negative blood cultures within seven days. This explains the complexity of diagnosing ICIs in the newborn and the need for a prompt empirical therapy at the time of diagnostic suspicion [92].

In particular, two new diagnostic molecular tools seem to be particularly promising to early diagnose ICIs, especially in the cases where a previous antifungal prophylaxis or empirical therapy could have reduced the possibility of a positive blood culture:

(a) the T2 Magnetic Resonance *Candida* Panel (T2 *Candida*, T2 Biosystems, Lexington, MA, USA) can detect five major *Candida* species (*C. albicans/C. tropicalis, C. parapsilosis,* and *C. krusei/C. glabrata*) directly in blood and it does not require viable organisms, with a lower time to positivity (lower than 3 h) [93,94]. T2 *Candida* can be used to efficiently diagnose or rule out candidemia even using low-volume blood specimens from pediatric patients: this could result in improved time to appropriate antifungal therapy or reduction in unnecessary empirical antifungal therapy [95].
(b) the indirect immunofluorescence assay (IFA) for *C. albicans* germ tube antibody (CAGTA) IgG is a method that enables the detection of specific IgG antibodies against antigens located on the cell wall surface of the mycelium of *Candida* spp. in human serum/plasma. Vircell Kit (Granada, Spain) and VirClia IgG Monotest (Granada, Spain) are the routine detection ways with widespread use in Europe. According to a systematic review, the diagnostic accuracy of the CAGTA assays is moderate for ICIs, and CAGTA findings should be interpreted in parallel with other biomarkers [96].

However, to best of our knowledge, there are still no studies in literature that evaluated the performance of these tests in the neonatal age only.

## 11. Treatment of Invasive *Candida* Infections

Mortality associated with *Candida* sepsis involves about half of infants, while survivors could develop severe long-term neurosensory impairment, including ocular, hearing, and cognitive impairment, cerebral palsy, and periventricular leukomalacia. Initial therapy is therefore often empirical, and the combination of prematurity, thrombocytopenia, and prolonged use of broad-spectrum antibiotics generally guides the initiation of empiric antifungal therapy [5].

Current Infectious Diseases Society of America guidelines recommend amphotericin B deoxycholate and fluconazole first-line therapies in infants with IC [44], while European guidelines recommend formulations of amphotericin B, fluconazole, and micafungin. Amphotericin B exists in various formulations, amphotericin B deoxycholate (D-AMPH-B), and liposomal amphotericin B (L-AMPH-B) [7]. The recommended dose for D-AMPH-B starts from 0.5–0.7 mg/kg/day up to 1.5 mg/kg/day. The recommended dose for L-AMPH-B is 3–5 mg/kg/day [97,98].

In neonates, the dose of fluconazole administered as therapy is 12 mg/kg/day regardless of birth weight or gestational age. The measurement of the blood levels reached (therapeutic drug monitoring) could help in establishing the drug concentrations actually reached during therapy. In fact, for many antifungal drugs, changes in clearance associa-ted with changes in birth weight and gestational age of newborns, especially preterm, have been observed [99]. In fact, in full-term infants, the plasma half-life of fluconazole is approximately 70 h (30 h in adults) while in premature infants it is 73 h at birth, 53 h at 6 days of age, and 46 h at 12 days of age. These pharmacokinetic characteristics make fluconazole a more attractive candidate for the prevention of ICI, mainly in premature infants, allowing for infrequent administration [100].

Although L-AMPH-B and D-AMPH-B are the most commonly used antifungal drugs in newborns, there are no prospective randomized neonatal studies that provide reliable information on the pharmacokinetic properties of these drugs and their safety.

All of these antifungals have unsatisfactory levels of evidence to support their use in neonates and, when used in this special patient population, they have limits ranging from renal and bone marrow toxicity to uncertain optimal dosage regimens, increased resistance of some *Candida* spp. and, finally, suboptimal spread to the kidneys or brain tissue. There is a need for alternative antifungal drugs with greater specificity and reduced toxicity in neonatal populations than those commonly used in the treatment of invasive neonatal infections.

Echinocandins could have a prominent role in contexts where there is a wide use of prophylaxis with fluconazole and resistance of *Candida* strains to azoles could emerge. Pharmacokinetic studies demonstrated excellent tolerability, safety, and efficacy of echinocandins in neonates. Furthermore, with their ability to target 1,3-beta-d-glucan synthesis as a means of inhibiting excess production of extracellular matrix, echinocandins represent an attractive therapy against *Candida* biofilms formation [101]. The in vitro efficacy of echinocandins in treating catheter biofilms was confirmed by Cateau et al., who found that lock solutions of 2 and 5 mg/L, respectively, of caspofungin and micafungin used to treat biofilms forming on a silicone catheter led to a significant and persistent reduction of yeast metabolic activity of intermediate and mature biofilms [102]. Additionally, biofilm impairment mediated by echinocandins would trigger a larger proinflammatory response from phagocytes, due to an increase in 1,3-beta-d-glucan exposure [103].

Some problems could emerge in the therapy against *Candida parapsilosis*. Echinocandins have in fact a high minimum inhibitory concentration against *Candida parapsilosis*. Despite this awareness, no clinical failures have been reported to date. Consequently, the resistance of *Candida parapsilosis* to echinocandins remains unexplored. Micafungin is the echinocandin with the more reliable evidence in neonatal population. It is the only echinocandin approved for neonates and young infants. The therapy at 8 mg/kg/day achieved a high response in a phase 2 study on 35 neonates with medical and surgical underlying diseases and confirmed or suspected ICIs [8].

## 12. Future Research Considerations

Prospective studies are needed to determine the clinical implications of new diagnostic molecular tools (T2MR and CAGTA) in neonatal age and their potential use in antimicrobial stewardship.

Empiric antifungal therapy needs further evidence sustaining the efficacy in reducing mortality and long-term neurodevelopmental disabilities in preterm infants and other categories of patients. Neonatal pharmacokinetics and pharmacodynamics data of the most-used antifungal drugs are still inconclusive, due to the complexity of carrying out this type of studies in the neonatal age. Furthermore, the clearing time of fungal infection in neonates and the microbiological criteria used to define clearance are currently ambiguous.

Concerning neonates with major surgical needs, admitted in NICUs, there is lack of a precise assessment of the incidence of fungal colonization and invasive infections and lack of evidence that may, or may not, support the benefits of antifungal prophylaxis. As of 9 January 2021, no trials on ICIs are enrolling infants after major surgery, according to clinical trial registries such as: https://clinicaltrials.gov (accessed on 9 March 2021) and https://www.umin.ac.jp/ctr (accessed on 9 March 2021).

Therefore, we are currently recruiting study subjects in a multicenter prospective observational study to assess the real incidence of ICIs in surgical neonates and infants up to three months of life in NICUs. The study involves 13 of the major Italian NICUs and it is coordinated by our group at Bambino Gesù Children’s Hospital (Rome, Italy). The primary outcome of the study is to assess the real incidence and risk factors of ICIs in neonates and infants up to three months of life requiring major surgery. We hope to provide the results of this research as soon as possible.

## 13. Conclusions

Infants requiring surgery carry many risk factors for candidemia and are likely to benefit from antifungal prophylaxis. To date, guidelines for the prevention of ICIs recommend intravenous or oral fluconazole prophylaxis in ELBW infants, while no specific recommendation is available for infants requiring major surgery. This finding should not be extrapolated from previous studies, and further epidemiologic data are needed to identify possible preventive strategies against candidemia in preterm and term infants who undergo major surgery.

## Figures and Tables

**Table 1:** Recent studies reporting prevalence of previous surgery in infants with invasive fungal infections.

Country and Year [Ref.]	Study Type	Study Period	Inclusion Criteria	Neonates (n)	Preterm (n, %)	Central Line (n, %)	Previous Surger (n, %)	Abdominal Surgery (n, %)	Non Abdominal Surgery (n, %)	In-Hospital Mortality (n, %)	Fungal Mortality (n, %)
US, 2000 [9]	R, SC	1981–1999	ICI	96	83 VLBW (86)	63 (66)	3 (12)	NA	NA	31 (32)	11 (11)
Kuwait, 2000 [10]	R, SC	1994–1998	Positive BC	25	9 LBW (36)	NA	25 (100)	20 (80)	5 (20)	8 (25)	NA
US, 2000 [11]	P, MC	1993–1995	Positive BC	35	29 VLBW (83)	NA	13 (37)	NA	NA	8 (23)	NA
Greece, 2004 [12]	P, SC	1994–2000	ICI	59	NA	NA	23 (39)	NA	NA	17 (29)	*C. albicans:* 15/38 (39)*; C. parapsilosis:* 1/9 (11)*;* others: NA
Costa Rica, 2005 [13]	R, SC	1994–1998	Positive BC	110	46 (62)	98 (89)	79 (72)	40 (36)	39 (35)	37 (34)	29 (26)
US, 2005 [14]	P, MC	1998–2000	Positive BC	35	30 VLBW (86)	23 (66)	13 (37)	8 (23)	5 (14)	7 (20)	NA
Jordan, 2008 [15]	R, SC	1995–2006	Positive BC	24	13 (54)	19 (79)	10 (42)	10 (42)	0	13 (54)	4 (17)
Australia, 2009 [16]	P, MC	2001–2004	Positive BC	33	33 (94)	24 (89)	5 (19)	NA	NA	7 (22)	NA
China, 2013 [17]	R, SC	2004–2010	IFI	45	29 VLBW (64)	32 (71)	NA	9 (20)	NA	4 (9)	NA
Portugal, 2014 [18]	P, MC	2005–2010	IFI	44	37 (84)	44 (100)	14 (32)	NA	NA	5 (11)	5 (11)
England, 2014 [19]	R and P, MC	R: 2004–2009; P: 2010	IFI	84	79 (94)	71 (87)	NA	11 (13)	NA	26 (31)	18 (21)
China, 2015 [20]	R, SC	2006–2010	Positive BC	19	NA	16 (84)	NA	9 (47)	NA	3 (16)	NA
Italy, 2016 [21]	P, MC	2005–2015	ICI	14	12 LBW (85)	NA	2 (14)	2 (14)	0	NA for neonates with ICI	NA for neonates with ICI
US, 2018 [22]	P, MC	2008–2015	Positive BC	90	46 (78)	70 (78)	8 (9)	2 (2)	7 (7)	14 (16)	NA
Iran, 2018 [23]	P, SC	2014–2016	Positive BC	35	17 (49)	33 (94)	14 (40)	10 (29)	4 (11)	15 (43)	NA for neonates with ICI
Germany, 2018 [24]	P, MC	2009–2015	Need for antifungal treatment	724	724 (100)	652 (90)	NA	272 (38)	NA	71 (10)	NA
Taiwan, 2018 [25]	R, SC	2004–2015	ICI	113	NA	108 (96)	31 (27)	NA	NA	48 (43)	32 (28)
Turkey, 2019 [26]	R, SC	2007–2012	ICI	22	20	3 (6)	5 (17)	NA	NA	10 (46)	NA
France, 2019 [27]	P, MC	2010–2012	IFI treated with micafungin	31	29 (97)	NA	NA	4 (4)	NA	0	0

BC: blood culture. ICI: invasive Candida infection. IFI: invasive fungal infection. LBW: low birth weight. MC: multi-center study. NA: not available. P: prospective. R: retrospective. SC: single-center study. VLBW: very low birth weight.

## References

[1] Hoyer L.L., Cota E. (2016). Candida albicans Agglutinin-Like Sequence (Als) Family Vignettes: A Review of Als Protein Structure and Function. Front. Microbiol..

[2] Filler S.G., Sheppard D.C. (2006). Fungal invasion of normally non-phagocytic host cells. PLoS Pathog..

[3] Staniszewska M. (2020). Virulence Factors in Candida species. Curr. Protein Pept. Sci..

[4] Hsieh E., Smith P.B., Jacqz-Aigrain E., Kaguelidou F., Cohen-Wolkowiez M., Manzoni P., Benjamin D.K. (2012). Neonatal fungal infections: When to treat?. Early Hum. Dev..

[5] Benjamin D.K., Stoll B.J., Gantz M.G., Walsh M.C., Sánchez P.J., Das A., Shankaran S., Higgins R.D., Auten K.J., Miller N.A. (2010). Eunice Kennedy Shriver National Institute of Child Health and Human Development Neonatal Research Network. Neonatal candidiasis: Epidemiology, risk factors, and clinical judgment. Pediatrics.

[6] Hope W.W., Castagnola E., Groll A.H., Roilides E., Akova M., Arendrup M.C., Arikan-Akdagli S., Bassetti M., Bille J., Cornely O.A. (2012). ESCMID* guideline for the diagnosis and management of Candida diseases 2012: Prevention and management of invasive infections in neonates and children caused by *Candida* spp.. Clin. Microbiol. Infect..

[7] Autmizguine J., Smith P.B., Prather K., Bendel C., Natarajan G., Bidegain M., Kaufman D.A., Burchfield D.J., Ross A.S., Pandit P. (2018). Fluconazole Prophylaxis Study Team. Effect of fluconazole prophylaxis on Candida fluconazole susceptibility in premature infants. J. Antimicrob. Chemother..

[8] Auriti C., Goffredo B.M., Ronchetti M.P., Piersigilli F., Cairoli S., Bersani I., Dotta A., Bagolan P., Pai M.P. (2021). High-dose micafungin in neonates and young infants with invasive candidiasis: Results of a phase 2 study. Antimicrob. Agents Chemother..

[9] Chapman R.L., Faix R.G. (2000). Persistently positive cultures and outcome in invasive neonatal candidiasis. Pediatric Infect. Dis. J..

[10] Mokaddas E., Ramadan S., Abo el Maaty S., Sanyal S. (2000). Candidemia in Pediatric Surgery Patients. J. Chemother..

[11] Saiman L., Ludington E., Pfaller M., Rangel-Frausto S., Wiblin R.T., Dawson J., Blumberg H.M., Patterson J.E., Rinaldi M., Edwards J.E. (2000). Risk factors for candidemia in Neonatal Intensive Care Unit patients. Pediatric Infect. Dis. J..

[12] Roilides E., Farmaki E., Evdoridou J., Dotis J., Hatziioannidis E., Tsivitanidou M., Bibashi E., Filioti I., Sofianou D., Gil-Lamaignere C. (2004). Neonatal candidiasis: Analysis of epidemiology, drug susceptibility, and molecular typing of causative isolates. Eur. J. Clin. Microbiol. Infect. Dis..

[13] Avila-Aguero M.L., Canas-Coto A., Ulloa-Gutierrez R., Caro M.A., Alfaro B., Paris M.M. (2005). Risk factors for Candida infections in a neonatal intensive care unit in Costa Rica. Int. J. Infect. Dis..

[14] Shetty S.S., Harrison L.H., Hajjeh R.A., Taylor T., Mirza S.A., Schmidt A.B., Sanza L.T., Shutt K.A., Fridkin S.K. (2005). Determining Risk Factors for Candidemia Among Newborn Infants from Population-Based Surveillance: Baltimore, Maryland, 1998–2000. Pediatric Infect. Dis. J..

[15] Badran E.F., Al Baramki J.H., Al Shamyleh A., Shehabi A., Khuri-Bulos N. (2008). Epidemiology and clinical outcome of candidaemia among Jordanian newborns over a 10-year period. Scand. J. Infect. Dis..

[16] Blyth C.C., Chen S.C., Slavin M.A., Serena C., Nguyen Q., Marriott D., Ellis D., Meyer W., Sorrell T.C. (2009). Australian Candidemia Study. Not Just Little Adults: Candidemia Epidemiology, Molecular Characterization, and Antifungal Susceptibility in Neonatal and Pediatric Patients. Pediatrics.

[17] Yu Y., Du L., Yuan T., Zheng J., Chen A., Chen L., Shi L. (2013). Risk factors and clinical analysis for invasive fungal infection in neonatal intensive care unit patients. Am. J. Perinatol..

[18] Baptista M.I., Nona J., Ferreira M., Sampaio I., Abrantes M., Tomé M.T., Neto M.T., Barroso R., Serelha M., Virella D. (2016). Invasive fungal infection in neonatal intensive care units: A multicenter survey. J. Chemother..

[19] Oeser C., Vergnano S., Naidoo R., Anthony M., Chang J., Chow P., Clarke P., Embleton N., Kennea N., Pattnayak S. (2014). Neonatal Infection Surveillance Network (neonIN). Neonatal invasive fungal infection in England 2004–2010. Clin. Microbiol. Infect..

[20] Liu M., Huang S., Guo L., Li H., Wang F., Zhang Q.I., Song G. (2015). Clinical features and risk factors for blood stream infections of Candida in neonates. Exp. Ther. Med..

[21] Mesini A., Bandettini R., Caviglia I., Fioredda F., Amoroso L., Faraci M., Mattioli G., Piaggio G., Risso F.M., Moscatelli A. (2016). Candida infections in paediatrics: Results from a prospective centre study in a tertiary care children’ s hospital. Mycoses.

[22] Benedict K., Roy M., Kabbani S., Anderson E.J., Farley M.M., Harb S., Harrison L.H., Bonner L., Wadu V.L., Marceaux K. (2018). Neonatal and Pediatric Candidemia: Results from Population-Based Active Laboratory Surveillance in Four US Locations, 2009–2015. J. Pediatric Infect. Dis. Soc..

[23] Charsizadeh A., Mirhendi H., Nikmanesh B., Eshaghi H., Makimura K. (2018). Microbial epidemiology of candidaemia in neonatal and paediatric intensive care units at the Children’s Medical Center, Tehran. Mycoses.

[24] Fortmann I., Hartz A., Paul P., Pulzer F., Müller A., Böttger R., Proquitté H., Dawczynski K., Simon A., Rupp J. (2018). German Neonatal Network. Antifungal Treatment and Outcome in Very Low Birth Weight Infants: A Population-based Observational Study of the German Neonatal Network. Pediatric Infect. Dis. J..

[25] Tsai M.H., Hsu J.F., Yang L.Y., Pan Y.B., Lai M.Y., Chu S.M., Huang H.R., Chiang M.C., Fu R.H., Lu J.J. (2018). Candidemia due to uncommon Candida species in children: New threat and impacts on outcomes. Sci. Rep..

[26] Öncü B., Belet N., Emecen A.N., Birinci A. (2019). Health care-associated invasive Candida infections in children. Med. Mycol..

[27] Leverger G., Timsit J.F., Milpied N., Gachot B. (2019). Use of Micafungin for the Prevention and Treatment of Invasive Fungal Infections in Everyday Pediatric Care in France: Results of the MYRIADE Study. Pediatric Infect. Dis. J..

[28] Ostrosky-Zeichner L., Sable C., Sobel J., Alexander B.D., Donowitz G., Kan V., Kauffman C.A., Kett D., Larsen R.A., Morrison V. (2007). Multicenter retrospective development and validation of a clinical prediction rule for nosocomial invasive candidiasis in the intensive care setting. Eur. J. Clin. Microbiol. Infect. Dis..

[29] Hermsen E.D., Zapapas M.K., Maiefski M., Rupp M.E., Freifeld A.G., Kalil A.C. (2011). Validation and comparison of clinical prediction rules for invasive candidiasis in intensive care unit patients: A matched case-control study. Crit. Care.

[30] Bassetti M., Righi E., Ansaldi F., Merelli M., Scarparo C., Antonelli M., Garnacho-Montero J., Diaz-Martin A., Palacios-Garcia I., Luzzati R. (2015). A multicenter multinational study of abdominal candidiasis: Epidemiology, outcomes and predictors of mortality. Intensive Care Med..

[31] Kett D.H., Azoulay E., Echeverria P.M., Vincent J.L. (2011). The EPIC II Group of Investigators. Candida bloodstream infections in intensive care units: Analysis of the extended prevalence of infection in intesive care unit study. Crit. Care Med..

[32] Kullberg B.J., Arendrup M.C. (2015). Invasive Candidiasis. N. Engl. J. Med..

[33] Vincent J.L., Rello J., Marshall J., Silva E., Anzueto A., Martin C.D., Moreno R., Lipman J., Gomersall C., Sakr Y. (2009). EPIC II Group of Investigators. International Study of the Prevalence and Outcomes of Infection in Intensive Care Units. JAMA.

[34] Autmizguine J., Hornik C.P., Benjamin D.K., Brouwer K.L., Hupp S.R., Cohen-Wolkowiez M., Watt K.M. (2016). Pharmacokinetics and Safety of Micafungin in Infants Supported with Extracorporeal Membrane Oxygenation. Pediatric Infect. Dis. J..

[35] Yan L., Yang C., Tang J. (2013). Disruption of the intestinal mucosal barrier in Candida albicans infections. Microbiol. Res..

[36] Manzoni P., Mostert M., Jacqz-Aigrain E., Stronati M., Farina D. (2012). Candida colonization in the nursery. J. Pediatric (Rio J.)..

[37] Mermel L.A., Allon M., Bouza E., Craven D.E., Flynn P., O’Grady N.P., Raad I.I., Rijnders B.J., Sherertz R.J., Warren D.K. (2009). Clinical Practice Guidelines for the Diagnosis and Management of Intravascular Catheter-Related Infection: 2009 Update by the Infectious Diseases Society of America. Clin. Infect. Dis..

[38] Dubbink-Verheij G.H., Bekker V., Pelsma I.C.M., van Zwet E.W., Smits-Wintjens V.E.H.J., Steggerda S.J., Te Pas A.J., Lopriore E. (2017). Bloodstream infection incidence of Different central Venous Catheters in Neonates: A Descriptive Cohort Study. Front. Pediatrics.

[39] Gominet M., Compain F., Beloin C., Lebeaux D. (2017). Central venous catheters and biofilms: Where do we stand in 2017?. APMIS.

[40] Karlowicz M.G., Hashimoto L.N., Kelly R.E., Buescher E.S. (2000). Should Central Venous Catheters Be Removed as Soon as Candidemia Is Detected in Neonates?. Pediatrics.

[41] Benjamin D.K., Stoll B.J., Fanaroff A.A., McDonald S.A., Oh W., Higgins R.D., Duara S., Poole K., Laptook A., Goldberg R. (2006). Neonatal Candidiasis Among Extremely Low Birth Weight Infants: Risk Factors, Mortality Rates, and Neurodevelopmental Outcomes at 18 to 22 Months. Pediatrics.

[42] Taylor J.E., Tan K., Lai N.M., McDonald S.J. (2015). Antibiotic lock for the prevention of catheter-related infection in neonates. Cochrane Database Syst. Rev..

[43] Pappas P.G., Kauffman C.A., Andes D.R., Clancy C.J., Marr K.A., Ostrosky-Zeichner L., Reboli A.C., Schuster M.G., Vazquez J.A., Walsh T.J. (2016). Clinical Practice Guideline for the Management of Candidiasis: 2016 Update by the Infectious Diseases Society of America. Clin. Infect. Dis..

[44] Bendel C.M., Wiesner S.M., Garni R.M., Cebelinski E., Wells C.L. (2002). Cecal Colonization and Systemic Spread of Candida albicans in Mice Treated with Antibiotics and Dexamethasone. Pediatric Res..

[45] Terrin G., Passariello A., De Curtis M., Manguso F., Salvia G., Lega L., Messina F., Paludetto R., Berni Canani R. (2012). Ranitidine is associated with infections, necrotizing enterocolitis, and fatal outcome in newborns. Pediatrics.

[46] Manzoni P., García Sánchez R., Meyer M., Stolfi I., Pugni L., Messner H., Cattani S., Betta P.M., Memo L., Decembrino L. (2018). Exposure to Gastric Acid Inhibitors Increases the Risk of Infection in Preterm Very Low Birth Weight Infants but Concomitant Administration of Lactoferrin Counteracts This Effect. J. Pediatrics.

[47] Austin P.D., Hand K.S., Elia M. (2016). Systematic review and meta-analyses of the effect of lipid emulsion on microbial growth in parenteral nutrition. J. Hosp. Infect..

[48] Caggiano G., Lovero G., De Giglio O., Barbuti G., Montagna O., Laforgia N., Montagna M.T. (2017). Candidemia in the Neonatal Intensive Care Unit: A Retrospective, Observational Survey and Analysis of Literature Data. Biomed. Res. Int..

[49] Becerra M.R., Tantaleán J.A., Suárez V.J., Alvarado M.C., Candela J.L., Urcia F.C. (2010). Epidemiologic surveillance of nosocomial infections in a Pediatric Intensive Care Unit of a developing country. BMC Pediatrics.

[50] Montagna M.T., Lovero G., De Giglio O., Iatta R., Caggiano G., Montagna O., Laforgia N. (2010). AURORA Project Group. Invasive fungal infections in Neonatal Intensive Care Units of Southern Italy: A multicentre regional active surveillance (Aurora Project). J. Prev. Med. Hyg..

[51] De Paula Menezes R., Silva F.F., Melo S., Alves P., Brito M.O., de Souza Bessa M.A., Amante Penatti M.P., Pedroso R.S., Abdallah V., Röder D. (2018). Characterization of Candida species isolated from the hands of the healthcare workers in the neonatal intensive care unit. Med. Mycol..

[52] Mahieu L.M., Van Gasse N., Wildemeersch D., Jansens H., Ieven M. (2010). Number of sites of perinatal Candida colonization and neutropenia are associated with nosocomial candidemia in the neonatal intensive care unit patient. Pediatric Crit. Care Med..

[53] Ramy N., Hashim M., Abou Hussein H., Sawires H., Gaafar M., El Maghraby A. (2018). Role of early onset neutropenia in development of candidemia in premature infants. J. Trop. Pediatrics.

[54] Cashen K., Reeder R., Dalton H.J., Berg R.A., Shanley T.P., Newth C., Pollack M.M., Wessel D., Carcillo J., Harrison R. (2018). Acquired infection during neonatal and pediatric extracorporeal membrane oxygenation. Perfusion.

[55] Manzoni P., Farina D., Leonessa M., Antonielli d’Oulx E., Galletto P., Mostert M., Miniero R., Gomirato G. (2006). Risk Factors for Progression to Invasive Fungal Infection in Preterm Neonates With Fungal Colonization. Pediatrics.

[56] Kühbacher A., Burger-Kentischer A., Rupp S. (2017). Interaction of Candida Species with the Skin. Microorganisms.

[57] Manzoni P., Farina D., Leonessa M., Priolo C., Arisio R., Gomirato G. (2007). Type and number of sites colonized by fungi and risk of progression to invasive fungal infection in preterm neonates in neonatal intensive care unit. J. Perinat. Med..

[58] Kaufman D.A., Gurka M.J., Hazen K.C., Boyle R., Robinson M., Grossman L.B. (2006). Patterns of Fungal Colonization in Preterm Infants Weighing Less Than 1000 Grams at Birth. Pediatric Infect. Dis. J..

[59] Robinson J.L., Davies H.D., Barton M., O’Brien K., Simpson K., Asztalos E., Synnes A., Rubin E., Le Saux N., Hui C. (2009). Characteristics and outcome of infants with candiduria in neonatal intensive care—A Paediatric Investigators Collaborative Network on Infections in Canada (PICNIC) study. BMC Infect. Dis..

[60] Yu J.C., Khodadadi H., Malik A., Davidson B., Salles É., Bhatia J., Hale V.L., Baban B. (2018). Innate Immunity of Neonates and Infants. Front. Immunol..

[61] Tsafaras G.P., Ntontsi P., Xanthou G. (2020). Advantages and Limitations of the Neonatal Immune System. Front. Pediatrics.

[62] Netea M.G., Joosten L.A., van der Meer J.W., Kullberg B.J., van de Veerdonk F.L. (2015). Immune defence against Candida fungal infections. Nat. Rev. Immunol..

[63] Maródi L., Johnston R.B. (2007). Invasive Candida species disease in infants and children: Occurrence, risk factors, management, and innate host defense mechanisms. Curr. Opin. Pediatrics.

[64] Maródi L. (2006). Innate cellular immune responses in newborns. Clin. Immunol..

[65] Naglik J.R., König A., Hube B., Gaffen S.L. (2017). Candida albicans—Epithelial interactions and induction of mucosal innate immunity. Curr. Opin. Microbiol..

[66] Naglik J.R., Gaffen S.L., Hube B. (2019). Candidalysin: Discovery and function in Candida albicans infections. Curr. Opin. Microbiol..

[67] Tong Y., Tang J. (2017). Candida albicans infection and intestinal immunity. Microbiol. Res..

[68] Richardson J.P., Moyes D.L., Ho J., Naglik J.R. (2018). Candida innate immunity at the mucosa. Semin. Cell Dev. Biol..

[69] Kaufman D., Fairchild K.D. (2004). Clinical microbiology of bacterial and fungal sepsis in very-low-birth-weight infants. Clin. Microbiol. Rev..

[70] Cleveland A.A., Farley M.M., Harrison L.H., Stein B., Hollick R., Lockhart S.R., Magill S.S., Derado G., Park B.J., Chiller T.M. (2012). Changes in Incidence and Antifungal Drug Resistance in Candidemia: Results from Population-Based Laboratory Surveillance in Atlanta and Baltimore, 2008–2011. Clin. Infect. Dis..

[71] Warris A., Pana Z.D., Oletto A., Lundin R., Castagnola E., Lehrnbecher T., Groll A.H., Roilides E., Andersen C.T., Arendrup M.C. (2020). EUROCANDY study group. Etiology and Outcome of Candidemia in Neonates and Children in Europe: An 11-year Multinational Retrospective Study. Pediatric Infect. Dis J..

[72] Steinbach W.J., Roilides E., Berman D., Hoffman J.A., Groll A.H., Bin-Hussain I., Palazzi D.L., Castagnola E., Halasa N., Velegraki A. (2012). International Pediatric Fungal Network. Results from a prospective, international, epidemiologic study of invasive candidiasis in children and neonates. Pediatric Infect. Dis. J..

[73] Hemedez C., Trail-Burns E., Mao Q., Chu S., Shaw S.K., Bliss J.M., De Paepe M.E. (2019). Pathology of Neonatal Non- albicans Candidiasis: Autopsy Study and Literature Review. Pediatric Dev. Patbol..

[74] Kaufman D.A. (2012). “Getting to Zero”: Preventing invasive Candida infections and eliminating infection-related mortality and morbidity in extremely preterm infants. Early Hum. Dev..

[75] Bassetti M., Marchetti M., Chakrabarti A., Colizza S., Garnacho-Montero J., Kett D.H., Munoz P., Cristini F., Andoniadou A., Viale P. (2013). A research agenda on the management of intra-abdominal candidiasis: Results from a consensus of multinational experts. Intensive Care Med..

[76] Baley J. (1991). Neonatal Candidiasis: The Current Challenge. Clin. Perinatol..

[77] Baley J., Kliegman R., Boxerbaum B., Fanaroff A. (1986). Fungal Colonization in the Very Low Birth Weight Infant. Pediatrics.

[78] Stoll B.J., Gordon T., Korones S.B., Shankaran S., Tyson J.E., Bauer C.R., Fanaroff A.A., Lemons J.A., Donovan E.F., Oh W. (1996). Late-onset sepsis in very low birth weight neonates: A report from the National Institute of Child Health and Human Development Neonatal Research Network. J. Pediatrics.

[79] Dodds Ashley E., Drew R., Johnson M., Danna R., Dabrowski D., Walker V., Prasad M., Alexander B., Papadopoulos G., Perfect J. (2012). Cost of Invasive Fungal Infections in the Era of New Diagnostics and Expanded Treatment Options. Pharmacotherapy.

[80] Harrington R., Kindermann S.L., Hou Q., Taylor R.J., Azie N., Horn D.L. (2017). Candidemia and invasive candidiasis among hospitalized neonates and pediatric patients. Curr. Med. Res. Opin..

[81] Schwab F., Geffers C., Piening B., Haller S., Eckmanns T., Gastmeier P. (2014). How many outbreaks of nosocomial infections occur in German neonatal intensive care units annually?. Infection.

[82] Qi L., Fan W., Xia X., Yao L., Liu L., Zhao H., Kong X., Liu J. (2018). Nosocomial outbreak of Candida parapsilosis sensu stricto fungaemia in a neonatal intensive care unit in China. J. Hosp. Infect..

[83] Guo W., Gu H.F., Zhang H.G., Chen S.B., Wang J.Q., Geng S.X., Li L., Liu P., Liu X., Ji Y.R. (2015). An outbreak of Candida parapsilosis fungemia among preterm infants. Genet. Mol. Res..

[84] Asadzadeh M., Ahmad S., Al-Sweih N., Hagen F., Meis J.F., Khan Z. (2019). High-resolution fingerprinting of Candida parapsilosis isolates suggests persistence and transmission of infections among neonatal intensive care unit patients in Kuwait. Sci. Rep..

[85] Kollef M., Micek S., Hampton N., Doherty J.A., Kumar A. (2012). Septic Shock Attributed to Candida Infection: Importance of Empiric Therapy and Source Control. Clin. Infect. Dis..

[86] Puig-Asensio M., Pemán J., Zaragoza R., Garnacho-Montero J., Martín-Mazuelos E., Cuenca-Estrella M., Almirante B. (2014). Prospective Population Study on Candidemia in Spain (CANDIPOP) Project, Hospital Infection Study Group (GEIH), Medical Mycology Study Group (GEMICOMED) of the Spanish Society of Infectious Diseases and Clinical Microbiology (SEIMC), & Spanish Network for Research in Infectious Diseases. Impact of therapeutic strategies on the prognosis of candidemia in the ICU. Crit. Care Med..

[87] Kaufman D., Boyle R., Hazen K.C., Patrie J.T., Robinson M., Donowitz L.G. (2001). Fluconazole prophylaxis against fungal colonization and infection in preterm infants. N. Engl. J. Med..

[88] Manzoni P., Stolfi I., Pugni L., Decembrino L., Magnani C., Vetrano G., Tridapalli E., Corona G., Giovannozzi C., Farina D. (2007). Italian Task Force for the Study and Prevention of Neonatal Fungal Infections, & Italian Society of Neonatology. A Multicenter, Randomized Trial of Prophylactic Fluconazole in Preterm Neonates. N. Engl. J. Med..

[89] Healy C.M., Baker C.J., Zaccaria E., Campbell J.R. (2005). Impact of Fluconazole prophylaxis on incidence and outcome of invasive Candidiasis in a Neonatal Intensive Care Unit. J. Pediatrics.

[90] Clerihew L., Austin N., McGuire W. (2007). Prophylactic systemic antifungal agents to prevent mortality and morbidity in very low birth weight infants. Cochrane Database Syst. Rev..

[91] Aliaga S., Clark R.H., Laughon M., Walsh T.J., Hope W.W., Benjamin D.K., Kaufman D., Arrieta A., Benjamin D.K., Smith P.B. (2014). Changes in the Incidence of Candidiasis in Neonatal Intensive Care Units. Pediatrics.

[92] Adams-Chapman I., Bann C.M., Das A., Ronald N., Stoll B.J., Walsh M.C., Sánchez P.J., Higgins R.D., Shankaran S., Watterberg K.L. (2013). Eunice Kennedy Shriver National Institutes of Child Health and Human Development Neonatal Research Network. Neurodevelopmental Outcome of Extremely Low Birth Weight Infants with Candida infection. J. Pediatrics.

[93] Patterson T.F., Donnelly J.P. (2019). New Concepts in Diagnostics for Invasive Mycoses: Non-Culture-Based Methodologies. J. Fungi..

[94] Mylonakis E., Clancy C.J., Ostrosky-Zeichner L., Garey K.W., Alangaden G.J., Vazquez J.A., Groeger J.S., Judson M.A., Vinagre Y.M., Heard S.O. (2015). T2 magnetic resonance assay for the rapid diagnosis of candidemia in whole blood: A clinical trial. Clin. Infect. Dis..

[95] Hamula C.L., Hughes K., Fisher B.T., Zaoutis T.E., Singh I.R., Velegraki A. (2016). T2Candida Provides Rapid and Accurate Species Identification in Pediatric Cases of Candidemia. Am. J. Clin. Pathol..

[96] Wei S., Wu T., Wu Y., Ming D., Zhu X. (2019). Diagnostic accuracy of Candida albicans germ tube antibody for invasive candidiasis: Systematic review and meta-analysis. Diagn. Microbiol. Infect. Dis..

[97] Pana Z.-D., Kougia V., Roilides E. (2015). Therapeutic strategies for invasive fungal infections in neonatal and pediatric patients: An update. Expert Opin. Pharm..

[98] Bradley J.S., Barnett E.D., Cantey J.B. (2019). Choosing Among Antifungal Agents: Polyenes, Azoles, and Echinocandins. Nelson’s Pediatric Antimicrobial Therapy.

[99] Scott B.L., Hornik C.D., Zimmerman K. (2020). Pharmacokinetic, efficacy, and safety considerations for the use of antifungal drugs in the neonatal population. Expert Opin. Drug Metab. Toxicol..

[100] Saxén H., Hoppu K., Pohjavuori M. (1993). Pharmacokinetics of fluconazole in very low birth weight infants during the first two weeks of life. Clin. Pharmacol. Ther..

[101] Larkin E.L., Dharmaiah S., Ghannoum M.A. (2018). Biofilms and beyond: Expanding echinocandin utility. J. Antimicrob. Chemother..

[102] Cateau E., Rodier M.H., Imbert C. (2008). In vitro efficacies of caspofungin or micafungin catheter lock solutions on Candida albicans biofilm growth. J. Antimicrob. Chemother..

[103] Katragkou A., Roilides E., Walsh T.J. (2015). Role of Echinocandins in Fungal Biofilm-Related Disease: Vascular Catheter-Related Infections, Immunomodulation, and Mucosal Surfaces. Clin. Infect. Dis..

